# Duchenne Muscular Dystrophy Newborn Screening: Evaluation of a New GSP^®^ Neonatal Creatine Kinase-MM Kit in a US and Danish Population

**DOI:** 10.3390/ijns5030027

**Published:** 2019-08-27

**Authors:** Anne Timonen, Michele Lloyd-Puryear, David M. Hougaard, Liisa Meriö, Pauliina Mäkinen, Ville Laitala, Tuukka Pölönen, Kristin Skogstrand, Annie Kennedy, Sari Airenne, Hanna Polari, Teemu Korpimäki

**Affiliations:** 1 PerkinElmer, Wallac Oy, Mustionkatu 6, 20750 Turku, Finland; 2 American College of Medical Genetics and Genomics, Bethesda, MD 20814, USA; 3 Danish Center for Neonatal Screening, Statens Serum Institut, 2300 Copenhagen, Denmark; 4 Parent Project Muscular Dystrophy, Hackensack, NJ 07601, USA

**Keywords:** Duchenne muscular dystrophy, creatine kinase, newborn screening

## Abstract

Duchenne muscular dystrophy (DMD/Duchenne) is a progressive X-linked disease and is the most common pediatric-onset form of muscular dystrophy, affecting approximately 1:5000 live male births. DNA testing for mutations in the dystrophin gene confirms the diagnosis of this disorder. This study involves assessment of screening newborns for DMD using an immunoassay for muscle-type (MM) creatine kinase (CK) isoform—the GSP Neonatal CK-MM kit. Comparisons were made with CK activity determination by fluorescence measurement. In addition, the study evaluated the effect of gestational age, age of infant at time of sampling and how stable the CK-MM was over time. This assay discriminates well between normal, unaffected and Duchenne affected populations and is suitable for Duchenne newborn screening.

## 1. Introduction

Duchenne muscular dystrophy (Duchenne/DMD) is an X-linked rare disease, affecting approximately 1:5000 live male births worldwide) [1,2]. DMD occurs as a result of mutations in the dystrophin gene that leads to an absence or reduction of the functional protein dystrophin and is manifested by progressive muscle degeneration [3]. Most patients are diagnosed at approximately 4–5 years of age, after their physical ability diverges from their peers. Clinically, DMD is characterized by progressive deterioration of muscle strength, and individuals with Duchenne typically require a wheelchair by their teens and have progressive upper limb weakness and cardiopulmonary involvement throughout their teen years and early 20s. Individuals with DMD typically die in their second decade due to cardiorespiratory failure [4,5,6,7].

Due to muscle degeneration, individuals with Duchenne have markedly elevated creatine kinase (CK) levels in blood. The elevation of CK in serum of DMD patients was first reported long ago [8,9] and detection of an elevated serum CK is currently one of the most common signs that trigger the suspicion of the diagnosis of muscle damage or a neuromuscular disorder [7,10,11].

Novel molecular and non-molecular therapies for treating Duchenne are under development [12]. Steroid therapy was first reported effective for boys with DMD in 1974 [13]. Since that time, many randomized controlled trials have shown that corticosteroids improve muscle strength and function for up to six months and strength up to two years [14]. More recent data from a randomized control trial suggest that corticosteroids produce better function over a five-year period in many patients [15], however, prolonged steroid use is associated with short stature and heavier weight. These growth alterations associated with steroid treatment should be considered when making treatment decisions for males with DMD [14].

One therapy (Ataluren), which targets nonsense mutations, has received conditional approval in Europe and in Australia for use in individuals with Duchenne. The U.S. Food and Drug Administration (FDA), has granted orphan drug designation to Ataluren for the experimental development of Duchenne. In September of 2016, FDA granted approval to the first Duchenne-specific drug therapy in the U.S. via the accelerated approval pathway to Eteplirsen, known commercially as EXONDYS 51, an exon skipping drug [16]. EXONDYS 51 targets mutations in a region implicated in 13% of DMD cases. The label for EXONDYS 51 allowed for broad access to patients amendable to an exon 51 skip of all ages. In February of 2017, the FDA approved a second Duchenne specific drug therapy named Deflazacort, known commercially as Emflaza. Emflaza is not mutation specific thus is amenable to all patients with Duchenne. Upon approval, the label for Emflaza included patients aged five and older and in June of 2019 was expanded to allow commercial access to patients as young as 24 months. In addition to the recently approved therapies, the Duchenne therapeutic pipeline includes more than 40 biopharmaceutical companies and nearly two dozen actively recruiting interventional trials representing a wide array of therapeutic approaches. The various approaches to treatment of Duchenne may be seen on the Parent Project Muscular Dystrophy website [12].

Historically, the primary approach to Duchenne newborn screening (NBS) has been through the detection of CK enzyme activity in serum by fluorescence measurement [17,18]. CK levels in newborns with Duchenne are significantly higher than the transiently elevated levels detected post birth indicating a cut-off-based approach to NBS was feasible [9]. This assay concept was converted for use with dried blood spot (DBS) samples in Duchenne NBS programs. Since 1975, ten Duchenne NBS programs [17] have provided opportunities to study screening protocols, outcomes, and parental responses. These programs used laboratory developed tests for detecting elevated CK enzyme activity levels in DBS for the initial screening, with the diagnosis of Duchenne based on findings of clinical follow-up, muscle biopsy, or direct mutational testing of the DMD gene. The programs screened more than 1.8 million newborns between 1975 and 2011, and 344 newborns were diagnosed with Duchenne. Across all programs, 80 patients had positive results for non-Duchenne disorders, including Becker muscular dystrophy (53 patients) and forms of limb-girdle and other congenital muscular dystrophies and 21 patients had false-negative findings for Duchenne. Three of these false negative findings were due to human error in the sample processing and reporting, and the remaining 18 cases were true false-negative findings with initial CK levels below each program’s cutoff [19]. To date, three new false negative cases have been identified resulting in a total of 24 patients with false-negative findings for Duchenne [20]. As more options for the treatment of Duchenne become available, refinement and interest towards Duchenne NBS is increasing.

Though the CK assay by fluorescence measurement works well with serum samples, it is not well suited for DBS [11]. Due to the DBS sample matrix, the enzyme activity assay is sensitive to background interference caused by endogenous ATP, a molecule present in most biological materials. Also, the volume of blood that is eluted from the DBS samples is significantly lower than what is typically the sample volume in the serum assays and so the DBS assays require more sensitivity than serum assays. Furthermore, several different forms of CK are found in blood (CK-MM, CK-MB, and CK-BB), all with the same enzyme activity. The CK-MM form that is found predominantly in skeletal muscles is the most specific marker of skeletal muscle damage caused by muscular dystrophies [11,19,21].

This paper presents an exploration of the use of a novel immunoassay to quantitatively determine CK-MM concentration in DBS to screen newborns for Duchenne. Comparisons were made with CK activity determination by fluorescence measurement. In addition, the study evaluated the effect of gestational age, age of infant at time of sampling and how stable the CK-MM was over time.

## 2. Materials and Methods

### 2.1. Study Populations: California and Denmark

Studies of the use of an immunoassay to detect Duchenne were performed at two different locations:

- Location 1: PerkinElmer, Wallac Oy in Finland using anonymous DBS samples from male and female newborns born in California, USA and obtained from the California Biobank Program;
- Location 2: Danish Center for Neonatal Screening, Statens Serum Institut (SSI), in Copenhagen, Denmark using anonymized DBS specimens obtained from Danish Neonatal Screening Biobank (DNSB or DNS-Biobank).

The DBS sample collection device was Whatman^®^ 903 filter paper. Archived, leftover specimens that had been stored in −20 °C before inclusion to the study were used. In addition to samples collected from presumed healthy newborns, samples from newborns that had been diagnosed later with Duchenne were included in the study at both locations to enrich the study populations. The Duchenne diagnoses had been confirmed by treating physicians and by molecular genetic testing.

### 2.2. Study Design

Location 1 studies were devoted to the evaluation of long-term sample stability, determining a preliminary range of CK-MM concentration in a US newborn population and establishing a preliminary cut-off threshold that would be used to categorize the samples as screen positives and negatives. In addition, the GSP CK-MM assay was compared to the fluorescent screening test method traditionally used to measure the CK activity [18]. Location 2 analyses were dedicated to producing a population distribution data for the CK-MM concentration in a Danish population and to determine the screening performance of the GSP^®^ Neonatal CK-MM kit.

The studies were obtained in accordance with requirements of study location and conducted in accordance with the Declaration of Helsinki, and Institutional Review Board (IRB) or ethic committee approvals or waivers for using anonymous DBS samples and associated data. The Committee for the Protection of Human Subjects, California Health and Human Services Agency (CPHS) had given the favorable opinion for the use of the specimens (Protocols #15-10-2260, 17 February 2016 and #15-02-1899, 21 September 2015) at Location 1. In addition, local IRBs at the clinical sites in California had given approvals or waivers to match the Duchenne diagnosis information to the Duchenne affected specimens archived in the biobank. The inclusion of DMD affected specimens required a signed consent by a parent or guardian based on approved protocol by the above-mentioned Institutional Review Boards (IRBs). The California Biobank Program gave a non-patient identifying specimen ID number to all specimens.

The specimens were included in the study at location 2 only if the parents/guardians of the newborn had not opted out the further use of the DBS used for NBS. The Danish ethics committee, De Videnskabsetiske Komiteer Region Hovedstaden, reviewed the study protocol and gave a waiver from ethics committee approval (protocol number 17011999, 27 April 2017). The Statens Serum Institut (SSI) gave a non-patient identifying specimen ID number all specimens.

### 2.3. Bloodspot CK-MM Concentration Analysis

The CK-MM concentration analysis was done using the GSP Neonatal Creatine Kinase -MM kit (cat. no. 3311-0010, For Investigational Use Only), DELFIA^®^ Inducer (cat. no. 3304-0010), Wash Concentrate (cat. no. 4080-0010), and fully automated GSP instrument (cat. no. 2021-0010) with software version 1.4 (all manufactured by PerkinElmer, Turku, Finland). The GSP CK-MM assay principle has been described by Moat et al. [11]. In short, the reagents included in the kit are calibrator paper cassettes, control paper cassettes with spots prepared from human blood enriched with human CK-MM, microtitration strips, tracer reagent, and a ready-for-use assay buffer. Calibrators, controls and DBS disks (3.2 mm/1/8-inch) are punched into the assay wells. Reagents and assay wells are loaded in the GSP instrument, after which the assay run starts and proceeds automatically.

The GSP CK–MM assay is a solid phase, 2-site immunofluorometric assay. CK-MM is eluted by the addition of the assay buffer and of the anti-CKMM- Europium tracer solution. CK-MM in the sample reacts with the immobilized mouse monoclonal antibodies and europium chelate–labeled mouse monoclonal antibodies, which recognize 2 separate antigenic sites on the molecular surface of CK-MM. After the incubation step with interval shaking, the paper disc and excess unbound label is washed away from the wells. Addition of the DELFIA^®^ inducer solution to the wells dissociates the europium ions from the tracer antibody attached label chelates into solution where they form highly fluorescent chelates. The fluorescence of each sample is proportional to the concentration of CK-MM.

### 2.4. Bloodspot CK Activity Analysis

The CK activity analysis measures the enzyme activity of CK in the DBS using fluorometry to determine the occurrence of DMD. The assay procedure is based on the reaction principle used by Orfanos and Naylor [18], employing a reactivation of CK activity with the addition of *N*-acetylacetyl-l-cysteine (NAC). CK catalyzes the transphosphorylation of ADP to ATP. Through a series of coupled reactions, the NADPH produced is measured at an excitation wavelength of 355 nm and an emission wavelength of 460 nm. The fluorescence is directly proportional to the NADPH concentration. The same calibrator and control material as for CK-MM concentration analysis were used for CK activity analysis (components of product cat. no. 3311-001, manufactured by PerkinElmer). The CK-activities of the calibrators were calculated based on the manufacturer’s reported activity of the pure CK-MM and the dilutions were made for each calibrator level. CK-NAC reagent was obtained from Thermo Scientific (cat. no. TR14010), Di (adenosine-5′) pentaphosphate (DAP) trilithium salt from Merck (cat. no. D6392) and fluorescence was measured with Victor2D Multilabel Counter (cat. no. 1420-020, PerkinElmer).

### 2.5. Calculations and Statistical Analysis

Statistical analyses were conducted with R, Spotfire S+ 8.1 and Minitab 18 software. The data was visualized with box plots and scatter plots and relevant descriptive statistics (sample size, mean, median value and range) were calculated. In addition, high percentiles (95%, 99% and 99.5%) were calculated and used as cut-off values in the study to classify the samples as screening positive and negative. Pearson and Kendall’s Tau correlation co-efficient were calculated and the relationship between the bloodspot CK-MM concentration analysis and CK activity was estimated using Passing–Bablok regression. Long term sample stability was evaluated using storage time specific population medians and visualizations.

## 3. Results

### 3.1. US Population and Long Term Sample Stability

**Figure 1:**
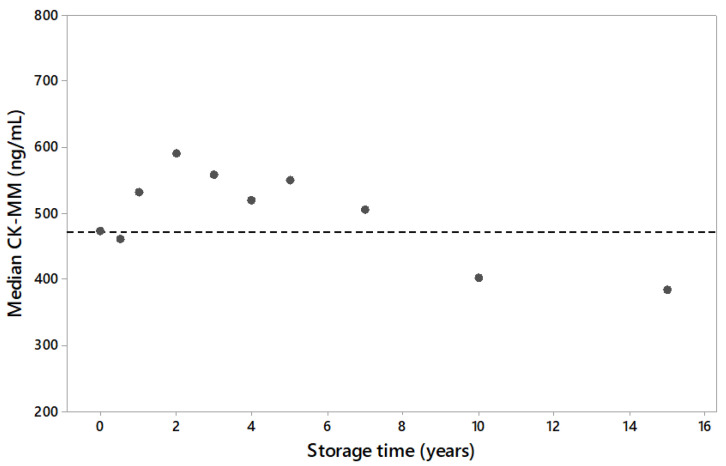
The stability of CK-MM in dried blood spot (DBS) specimens stored in a freezer (−20 °C) for a long period of time (years) presented as population median by storage time. A total of 120 sample results per time-point. Dotted line represents the median value of first timepoint.

There were two different sets of DBS samples from a USA population used. The first DBS set included 1200 samples (120 per storage time point, timepoints 0, 6 months, 1, 2, 3, 4, 5, 7, 10, 15 years) to determine the long-term stability of CK-MM in DBS when stored in a freezer (−20 °C). The CK-MM concentration across archived samples were comparable to fresh samples up to 7 years of storage time, and that variability within any given storage time point was similar between archived time points and fresh samples. The population median of 10- and 15-year timepoints showed a smaller CK-MM concentration in DBS samples (Figure 1).

Second set of DBS samples included 721 anonymous newborn samples of which 19 had been affected by Duchenne, one sample was from a Duchenne carrier and one from a Becker carrier. Samples had been stored for approximately 7–12 years before the bloodspot CK-MM concentration analysis. The storage times of affected and unaffected samples were matched. The age at the time of initial specimen collection varied between 12 and 61 h of age and the birth weight varied between 780 and 5555 g. Three hundred thirty-two (47%) of 700 samples were collected from females and 368 (53%) from males. The CK-MM concentration method was compared to the CK activity method. Same quality controls were run with both methods (Table 1).

**Figure 2:**
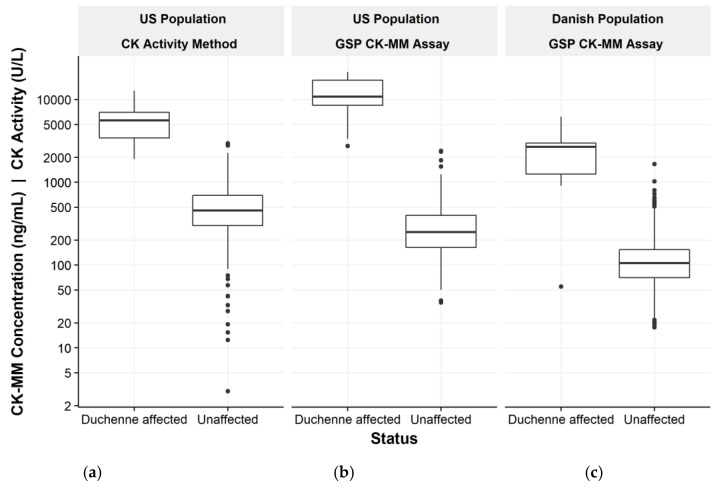
CK activity (U/L) box plot (**a**) and CK-MM concentration (ng/mL) box plot (**b**) of presumed negative (*n* = 700) and confirmed positive newborn specimens (*n* = 19) from US population. The highest Duchenne affected samples had CK-MM concentrations above the measuring range of the assay (6.8 to 8000 ng/mL). CK-MM concentration (ng/mL) box plot (**c**) present the presumed negative (*n* = 1408) and confirmed positive newborn specimens (*n* = 16) from Danish population. CK-MM concentration was not elevated in one Duchenne positive sample taken from and preterm newborn with low birth weight (Danish population).

Nineteen of the Duchenne affected samples were from males. In addition, one female Duchenne carrier and one female Becker carrier samples were tested. The CK-MM concentrations measured from the Duchenne affected specimens were within a range (2750–21,600 ng/mL) that was fully above the range of the unaffected population. However, the Duchenne carrier result was within the normal range (197 ng/mL) whereas the Becker carrier (2100 ng/mL) separated well from the unaffected population with both cutoff values. All Duchenne affected specimens were detected with the GSP CK-MM kit using the 99th percentile cut-off, whereas the CK enzyme activity method missed one affected Duchenne specimen (Table 2, Figure 2). The results of GSP CK-MM method were compared to the enzymatic CK activity method and showed a significant linear correlation (Pearson correlation of 0.934, *p* < 0.001).

### 3.2. Danish Population

The Danish population included data for 3526 anonymous newborn samples of which 16 had been affected by DMD, and three had been affected by BMD. Two thousand ninety-nine of unaffected specimens were used to determine testing site-specific newborn population distribution and higher percentile based cutoff values (Table 3).

**Figure 3:**
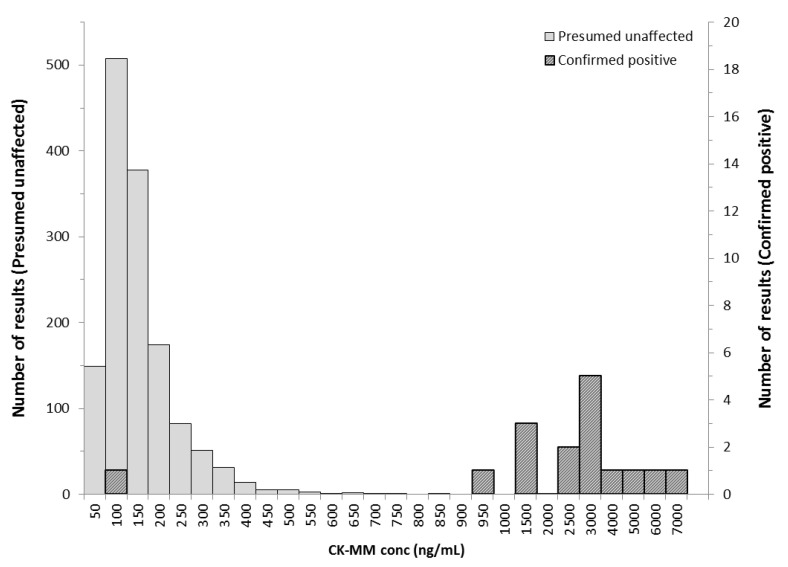
CK-MM concentration (ng/mL) of distribution of presumed negative (*n* = 1408) and confirmed positive newborn specimens (*n* = 16). CK-MM concentration was not elevated in one Duchenne positive sample taken from and preterm newborn with low birth weight (Danish population).

The next 1408 unaffected specimens together with 16 retrospective confirmed Duchenne positive samples were assayed and categorized as screening positive or negative based on the cutoff values. The mean value for presumed unaffected specimens was 128 ng/mL and for Duchenne affected 2626 ng/mL. With 99.5th percentile cut-off value, 15 of the Duchenne affected specimens were classified as screening positive and one specimen as screening negative. The specimen had been collected from a premature newborn (gestational age of 27 weeks and birth weight 1200 g) when the newborn was 3 days old. The repeat specimen (collected at age of 35 days) was analyzed and CK-MM was higher but not elevated above the unaffected population. Kit discriminated between the normal population and screening positive DMD cases with a positive predictive value (PPV) of 93.8% (Table 4 and Table 5, Figure 2 and Figure 3). In addition, four specimens where excluded from the primary data analysis as they did not meet the intended use of the GSP CK-MM assay or study inclusion criteria. These specimens included one Duchenne female carrier, and four samples from Becker cases. All these samples had a screening negative result.

### 3.3. Affect of the Age and Gestational Age of the Newborn

**Figure 4:**
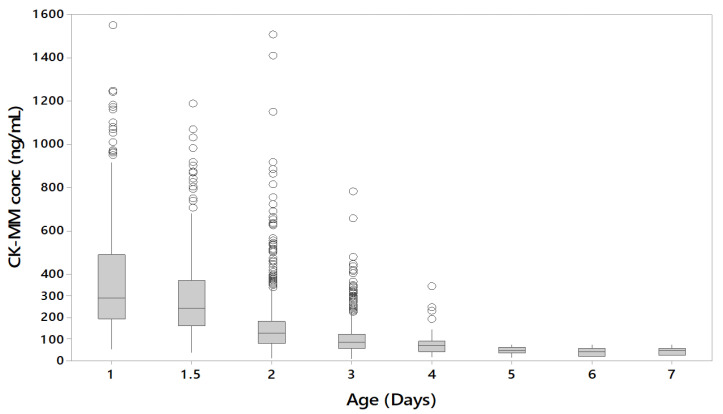
CK-MM concentration (ng/mL) by age (unaffected samples from US and Danish populations).

The data from the unaffected samples of US and Danish populations were combined to evaluate the effect of the age of the newborn at the time of sampling to the CK-MM concentration levels. The US population samples had been collected during the first 1–2 days (24–48 h) after birth and most of the Danish population samples were collected at 2–3 days of age or later. The CK-MM concentration was found to inversely correlate with the age of the newborn in unaffected specimens (Figure 4).

**Figure 5:**
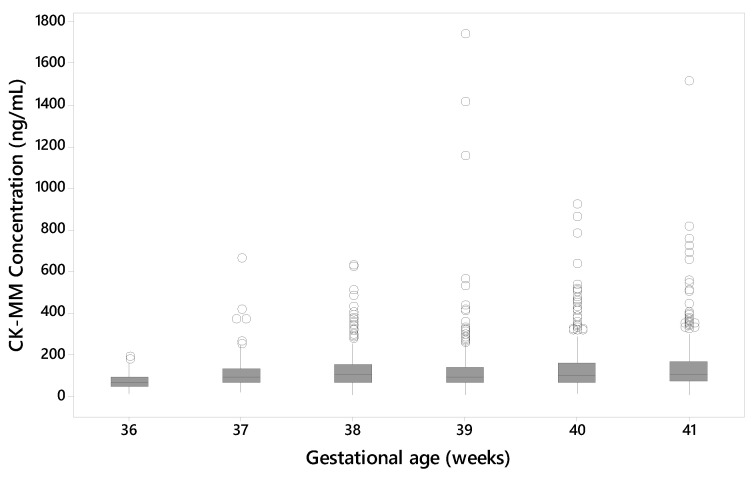
CK-MM concentration (ng/mL) by gestational age (unaffected samples from Danish population).

The data from the unaffected samples of Danish populations was used to evaluate the effect of the gestational age of the newborn to the CK-MM concentration levels. The median CK-MM concentration was increased with the gestational age of the newborn in unaffected specimens (Figure 5).

## 4. Discussion

In NBS, determination of presumptive positives for Duchenne would use a cut-off value threshold to distinguish between presumptive negative and presumptive positive values. The cut-off value would be determined based on the distribution of creatine kinase in the newborn population. Blood collected from a heel prick with direct application onto the filter paper is the preferred sample for NBS. The study demonstrated that the novel GSP CK-MM assay discriminates well between normal, unaffected and Duchenne affected populations and is suitable for Duchenne NBS from DBS. In addition, unlike previous methodologies for detecting CK enzymatic activity, the CK-MM concentration analysis shows improved specificity.

As reported by Moat et al. [11], humidity and temperature have significant effect on the stability of CK-MM in DBS samples. For short term, samples could be stored at room temperature or lower dry conditions. Acceptable long-term stability is achieved using desiccants and freezing DBS samples at −20 °C. Storage of samples in an environment with elevated temperatures and/or humidity can increase the risk of false negative results. In the present study, archived leftover specimens had been stored in −20 °C before inclusion to the study. To confirm the integrity of archived samples, long-term sample stability study was done. The study showed degradation resulting in the decrease of the CK-MM concentration in DBS samples stored longer than 7 years as frozen. Because Duchenne causes the CK-MM to elevate, the sample degradation was considered when the subsequent studies were planned. The storage time of affected and unaffected samples was either matched to be the same or the unaffected samples were stored for shorter times than the affected samples. This arrangement prevented the studies from overestimating the efficacy of CK-MM due to sample degradation, since the Duchenne affected samples would be equally or more degraded than the unaffected samples. Thus, using archived samples would cause, in the worst case, an underestimation of the CK-MM concentration method screening efficacy.

One of the Duchenne affected newborns that was included in the two studies was collected from a preterm (gestational age 27 weeks) baby with low birth weight (1200 g) resulting in a false negative result (55.2 ng/mL). The median CK-MM concentration also increased with the gestational age of the newborn in unaffected specimens. Another affected newborn, with a gestational age of 35 weeks and birth weight of 2700 g had a CK-MM concentration of 1100 ng/mL i.e., above the used cutoff values. Previous prenatal studies [22] have showed that some affected fetuses do not have elevation in CK. Since the CK-MM is primarily a marker of skeletal muscle damage and thus an indirect marker of Duchenne, Duchenne affected infants may be born who have not yet sustained enough muscle damage to elevate the CK-MM marker above the cut-off level. Duchenne NBS programs might benefit from using a different screening algorithm for low birth weight and preterm newborns as recommended by CLSI guideline NBS03 [23].

Duchenne and Becker muscular dystrophies are considered a spectrum of the same disease, having similar signs and symptoms and being caused by different mutations in the same gene. Both Duchenne and Becker are X-linked recessive diseases, so females are typically asymptomatic carriers of mutations. However, female carriers may manifest symptoms varying from mild muscle weakness to a more severe clinical course and are classified as manifesting or symptomatic carriers [24]. Female carriers or individuals with Becker having elevated CK-MM concentrations at birth may result as screen positive (depending on the cutoff value used) in any NBS program for Duchenne. Blood CK-MM is primarily a marker of skeletal muscle damage and thus an indirect marker of Duchenne which causes skeletal muscle damage. As noted during the earlier Duchenne NBS programs [25], other conditions causing muscle damage, such as forms of limb-girdle and other congenital muscular dystrophies may be detected as screen positives.

In our study, one of the specimens collected was from a female subject, who was a manifesting Duchenne carrier. Both the initial and repeat CK-MM results were screen negative with both cut-offs used. Also, the enzymatic CK activity method result was screen negative. In contrast, a specimen that had been collected from her twin brother, with the same dystrophin gene mutation and the typical Duchenne phenotype, measured as screen positive with both cut-offs and both methods.

In addition to Duchenne carriers, Becker muscular dystrophy samples were analyzed. Becker is an X-linked disorder caused by non-truncating dystrophin gene mutations, leading to altered, but detectable dystrophin expression in muscle fibers. Becker is typically characterized by later-onset skeletal muscle symptoms than Duchenne although the phenotype can vary. In our study, one specimen collected from a Becker carrier was analyzed resulting in a screen positive result and four specimens collected from Becker cases had a screen negative result. Some individuals with Becker may have elevated CK-MM concentrations at birth and may screen positive [26].

The CK-MM concentration was found to inversely correlate with the age of the newborn in unaffected specimens. CK-MM is typically highly elevated in Duchenne patients, however during the first few days after birth CK-MM may also be elevated in unaffected newborns due to muscle trauma during birth [9]. After birth, the CK-MM levels of unaffected newborns decline, whereas patients with muscular dystrophies continue to have elevated CK-MM levels. Therefore, it is recommended that laboratories should take this into account when establishing their cutoffs.

Since previous pilot Duchenne NBS programs had used creatine kinase enzymatic activity methods, a comparison of the two methods was done. A significant association between the CK-MM concentration and CK enzymatic activity was observed, which further supported the suitability of the CK-MM for Duchenne screening in newborns. The correlation has also been demonstrated previously with 10 blood spot samples from older boys with DMD [11]. However, all Duchenne affected specimens were detected with the GSP CK-MM kit using the 99th percentile cut-off, whereas the enzymatic activity method missed one of the affected Duchenne specimens. The novel GSP CK-MM assay discriminates better between normal, unaffected and Duchenne affected populations.

## Figures and Tables

**Table 1:** Quality control performance data with GSP CK-MM concentration analysis and creatine kinase (CK) enzyme activity analysis. Same quality controls were run with both method.

Control	*n*	GSP CK-MM Assay		CK Activity Method	
		Mean (ng/mL)	CV%	Mean (U/L)	CV%
QC1	22	119.1	7	463.4	17
QC2	22	417.2	7	685.9	14
QC3	22	1863.2	8	1845.2	9

**Table 2:** Range and mean for Duchenne affected and unaffected newborn DBS with GSP CK-MM concentration analysis and CK enzyme activity analysis and cutoff used to compare the screening performance of the two methods.

Sample	GSP CK-MM Assay (ng/mL)						CK Activity Method (U/L)				
	*n*	Min	Max	Mean	95th	99th	Min	Max	Mean	95th	99th
Duchenne affected	19	2750	21,600	12,144			1910	12,800	5822		
Unaffected	700	35.2	2390	328	867	1190	0	2950	547	1300	1980

**Table 3:** Range mean and median for unaffected newborn DBS with GSP CK-MM concentration (ng/mL) analysis (Danish population).

*n*	Mean	Median	Min	Max	95th	99th	99th
2099	124	96.6	<6.8	1740	291	513	675

**Table 4:** Range, mean and median for Duchenne affected and unaffected newborn DBS with GSP CK-MM concentration (ng/mL) analysis.

Sample	*n*	Mean	Median	Min	Max
Duchenne affected	16	2626	2685	909 ^1^	6230
Unaffected	1408	128	106	17.7	1660

^1^ CK-MM concentration of a preterm newborn with low birth weight was 55.2 ng/mL.

**Table 5:** Screening performance of GSP CK-MM immunoassay using 99.5th percentile (cut-off 675 ng/mL). Overall percent agreement was 99.6% (CI 99.2–99.9%), positive percent agreement 93.8% (CI 69.8–99.8%) and negative percent agreement = 99.7% (CI 99.3–99.9%).

Screening Result	Clinical Status		Total
	Duchenne Affected	Unaffected	
Screening positive	15	4	19
Screening negative	1 ^1^	1404	1405
TOTAL	16	1408	1424

^1^ CK-MM concentration was not elevated in one Duchenne affected sample taken from a preterm newborn with low birth weight.
